# A novel state space reduction algorithm for team formation in social networks

**DOI:** 10.1371/journal.pone.0259786

**Published:** 2021-12-02

**Authors:** Muhammad Zubair Rehman, Kamal Z. Zamli, Mubarak Almutairi, Haruna Chiroma, Muhammad Aamir, Md. Abdul Kader, Nazri Mohd. Nawi

**Affiliations:** 1 Faculty of Computing and Information Technology, Sohar University, Sohar, Sultanate of Oman; 2 Faculty of Computing, Universiti Malaysia Pahang (UMP), Gambang, Pahang, Malaysia; 3 College of Computer Science and Engineering, University of Hafr Al Batin, Hafar Al-Batin, Saudi Arabia; 4 Abubakar Tafawa Balewa University, Bauchi, Nigeria; 5 University of Derby, Derby, United Kingdom; 6 Soft Computing and Data Mining Centre (SMC), Faculty of Computer Science & Information Technology, Universiti Tun Hussein Onn Malaysia (UTHM), Batu Pahat, Malaysia; Torrens University Australia, AUSTRALIA

## Abstract

Team formation (TF) in social networks exploits graphs (i.e., vertices = experts and edges = skills) to represent a possible collaboration between the experts. These networks lead us towards building cost-effective research teams irrespective of the geolocation of the experts and the size of the dataset. Previously, large datasets were not closely inspected for the large-scale distributions & relationships among the researchers, resulting in the algorithms failing to scale well on the data. Therefore, this paper presents a novel TF algorithm for expert team formation called SSR-TF based on two metrics; communication cost and graph reduction, that will become a basis for future TF’s. In SSR-TF, communication cost finds the possibility of collaboration between researchers. The graph reduction scales the large data to only appropriate skills and the experts, resulting in real-time extraction of experts for collaboration. This approach is tested on five organic and benchmark datasets, i.e., UMP, DBLP, ACM, IMDB, and Bibsonomy. The SSR-TF algorithm is able to build cost-effective teams with the most appropriate experts–resulting in the formation of more communicative teams with high expertise levels.

## Introduction

Since the beginning of time, the human race has collaborated and coordinated on activities that are deemed impossible for one human to execute independently. The collaboration on these activities has always been highly influenced by geography and location constraints. In the past, the teams were created based on the individuals present in the same vicinity. This practice resulted in the team formation of individuals who lacked the necessary skills to execute the project successfully [1]. Being an operation’s research problem, team formation (TF) effectively selects qualified members for software project management, community collaboration, social networks, etc. More recently, TF is used for selecting team members in a social network graph, in which each individual is represented by a node and has some skills and can connect according to some edge weights to fulfill a task [2, 3]. Seeing this possibility, social networks between individuals have become a norm among people working in the same company. In fact, successful team collaborations have emerged between the people of the same department [4]. The advent of the cyber age has nullified location and geographical constraints. With high-speed fifth generation, mobile broadband, and fast traveling, the possibility of gaining a qualified person’s knowledge and expertise has become relatively easy.

Moreover, the internet has also brought individuals possessing different skills with the same interests to come together and team through social research networks like ResearchGate and Mendeley. In 2009, Lappas et al. tackled team formation and tried to find expert teams that can fulfill all tasks with minimum communication cost. They called TF an NP hard problem because no polynomial-time algorithm has been able to solve it [4, 5]. Existing approaches tried to identify teams with minimum communication costs, balanced workloads, personnel costs, and team reliability, unique experts, or all of them combined. The summary of all the popular TF algorithms is given in Table 2, where it can be noted that all the current works were beneficial to an extent. However, these approaches did not reduce the size of the search space required to fulfill a task, thus failing to scale well on large datasets. Therefore, this research will reduce the search space in breadth and depth so that effective teams within polynomial time can be formed. The contributions of the research are listed as follows;

- One-hot encoding machine learning scheme is applied for the first time in team formation problem in SSR algorithm during the binarization process of skills. One-hot encoding is used to label the skills as present (1) or absent (0) [6]. This led to the faster execution of the algorithm over binary data.
- One-hot encoding helps in realizing the edges with or without weights. The removal of zero weighted edges resulted in a reduced graph with only the required skills or features.
- The SSR algorithm has shown polynomial-time during convergence when tested on organic/benchmark datasets against state-of-the-art metaheuristic algorithms.

The following section explains the Team formation problem in social networks, followed by the related work on team formation, the performance of the improved algorithm on a real dataset from the Association for Computing Machinery (ACM) is discussed along with a case study, then proposed methodology along with the simulations results is presented, and finally the paper is concluded with discussions on the simulation results generated by the proposed SSR-TF and the comparison algorithms.

## Team formation in social networks

The Team Formation (TF) problem is defined as the minimization of two objectives: the communication cost and the search space, to form an effective team that can perform all the required tasks. The terms and mathematical notations are given in Table 1.

**Table 1:** Terminologies and notations.

Notation	Meaning	Notation	Meaning
**ACM**	Association for Computing Machinery	**MBTI**	Myer-Briggs Type Indicators
**BRADO**	Brain Drain Optimization	**MST**	Minimum Spanning Tree
**CC**	Communication cost between two experts	**NSGA-II**	Non-Dominated Sorting Genetic Algorithm
**D01**	Dataset 01	**PSO**	Particle Swarm Optimization
**D02**	Dataset 02	***S***	Number of skills defined in social network
**D03**	Dataset 03	***S’***	A set of reduced number of skills in social network
**D04**	Dataset 04	***SSR-TF***	Search Space Reduction Team Formation
**D05**	Dataset 05	***s(x***_***i***_ ***)***	An expert *x*_*i*_ having a skill
**DBLP**	Database Systems & Logic Programming	***SSR***	State Space Reduction / Search Space Reduction
***d*(*TE***_***i***_ **, *TE***_***j***_ **)**	The shortest distance between the two experts with skills	***SP(s***_***k***_ ***)***	A set of professionals skilled in *s*_*k*_
**G**	Graph of experts with number of skillsets	***T***	Tasks to be fulfilled by experts of specific skills
**G’**	Sub-graph of experts with required skillsets	***TC***	The sum of distance between every expert pair in the team
**GA**	Genetic algorithm	**TE**	A Team of Experts
**IMDB**	Internet Movie Database	**TF**	Team formation
**IPD**	integrated product development	**UMP**	Universiti Malaysia Pahang
**ChemoTF**	Chemistry Oriented Team Formation	***X***	Number of experts in social network
**IABO**	Improved African Buffalo Optimization	***X’***	A set of reduced unique experts in the team
**MBPSO/MOPSO**	multi-objective particle swarm optimization	***XS***	person *X* with their particular skillsets

### Problem 1

Team formation can be considered a graph, *G*(*X*,*S*) consisting of m number of experts, *X* = {*x*_1_,…,*x*_*m*_} and n number of skills *S* = {*s*_1_,…,*s*_*n*_}. Each expert *x*_*i*_ has a set of skills, *s*(*x*_*i*_)⊆*S*, then the set of the skilled expert with skills *s*_*k*_ is denoted by *SP*(*s*_*k*_)⊆*X*. The Task T tries to find all the experts *x*_*i*_ that cover all/some of the skills belonging to set *S* [7].

1. **Communication Cost (CC):** is the measure of how closely related two experts are in the given social network based on their common skills. The CC between the two adjacent experts (*x*_*i*_, *x*_*j*_) in graph *G*(*X*,*S*) is calculated with Jaccard distance as given in Eq (1). Meanwhile, the CC between non-adjacent experts (*x*_*i*_, *x*_*j*_) is the sum of the shortest path between them.

$$CC(x_{i},x_{j})=1-\frac{s(x_{i})\cap s(x_{j})}{s(x_{i})\cup s(x_{j})}$$ (1)

Total Cost (TC) is the measure of the total distance between a Team of Experts (TE) with skills from graph *G*(*X*,*S*) and defined as [8];

$$TC=\sum_{i}^{n}\sum_{j=i+1}^{n}d({TE}_{i},{TE}_{j})$$ (2)

**Fig 1:**
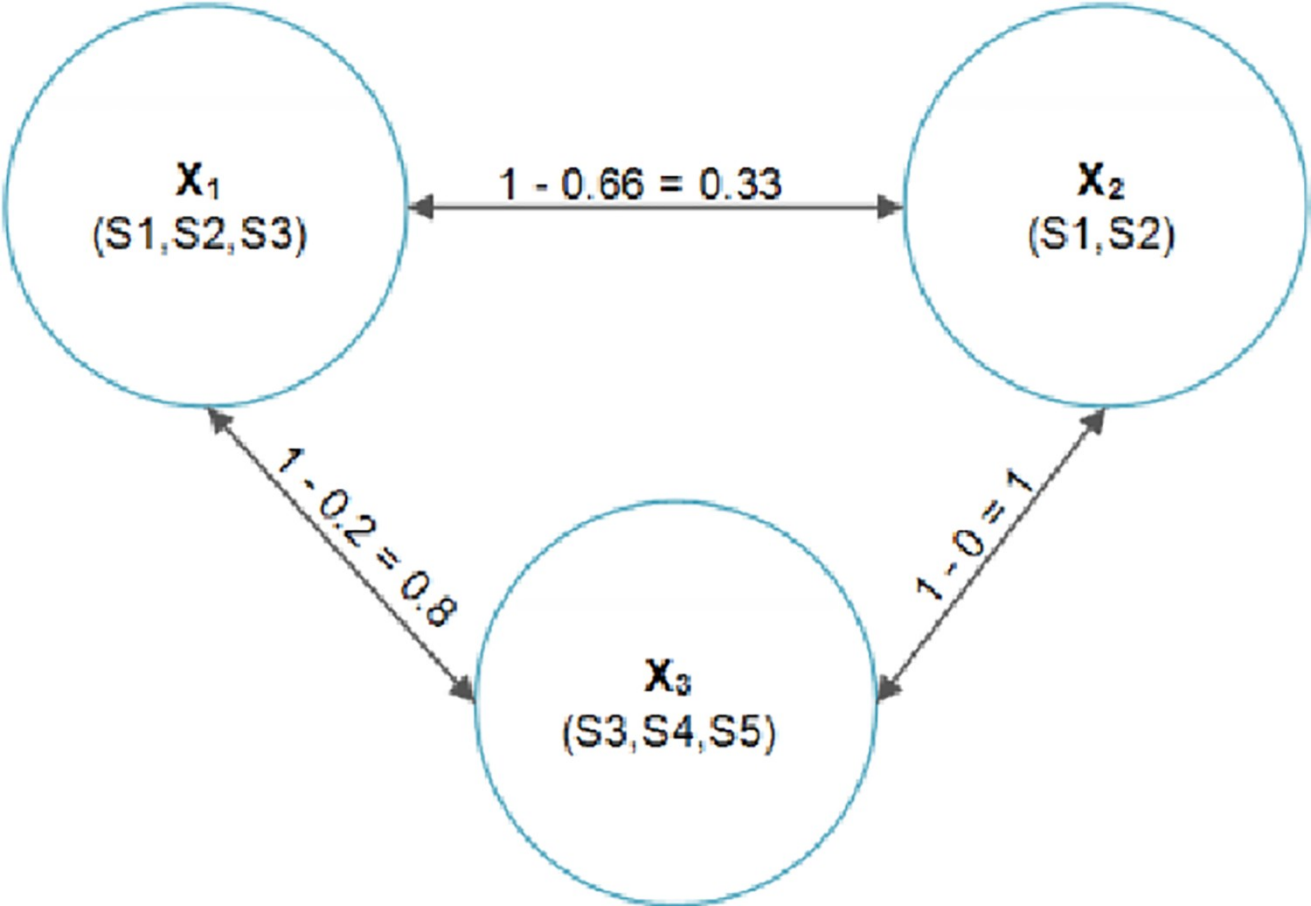
An example of team formation in social networks.

Fig 1 shows the possible team formation of three experts *X* = {*x*_1_, *x*_2_, *x*_3_} with respect to the connection cost based on five skills *S* = {*s*_1_, *s*_2_, *s*_3_, *s*_4_, *s*_5_}. For example, *TE* can be formed for the required skills {*s*_1_, *s*_2_, *s*_4_}. Forming a social network of teams is to reduce the communication cost between all the experts. Here, some of the possible teams are *T*_1_ = {*x*_1_, *x*_3_} and *T*_2_ = {*x*_2_, *x*_3_}. The goal of the proposed heuristic algorithm is to find the least communication cost among all team members.

2. **Search Space Reduction:** To reduce the search space, a sub-set of original data was obtained, which was able to represent the original set of data. The data was reduced both horizontally (selecting skills only required in the given task) and vertically (discarding experts not having any required skills for a given task) to obtain the sub-search space [9, 10]. Ultimately a reduced graph *G*′ is generated, which contains reduced experts *X*′ and reduced skills *S*′. The optimal solutions are then searched in the reduced graph *G*′(*X*′,*S*′).

## Related work

**Fig 2:**
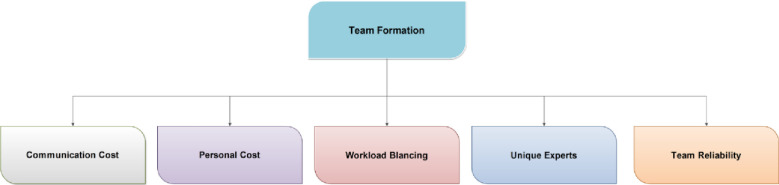
Common team formation attributes in social networks.

Since its inception, Team formation is considered solely dependent on the communication cost. With the passage of time, attributes like personal cost [1], workload balancing [11], unique experts [8, 12], and team reliability [13, 14] were also added by the researchers to create teams according to their needs. Team formation attributes are given in the Fig 2 and the TF algorithms are discussed based on all or some of these attributes subsequently.

Extensive work has been done by the Operations research community on Team Formation (TF) in which they have considered it as a linear integer problem (LIP) and focused entirely on finding links between people and the required functional skills [4, 15]. In 2009, Lappas et al. [4] introduced TF as a graph to the data mining community and considered the minimum cost of communication between the social network of experts. They utilized search heuristic functions to approximate the communication costs of the team. The radius function used by them finds the longest shortest path between the two experts, and the Enhanced-Steiner used the minimum-spanning tree (MST) cost of the sub-graph. Nevertheless, both methods were insensitive to adding or deleting a connection in the graph, thus bringing a radical change to the solution [3]. The same year, Abdelsalam suggested using multi-objective particle swarm optimization (MBPSO) algorithm for efficient team formation in integrated product development (IPD) for complex environments. The problem was broken into three parts: (1) team formation by collecting individuals with specific skills; (2) to ensure team efficiency Myer-Briggs Type Indicators (MBTI) was used for an individual’s personality profiling. MBTI helped create teams with people of the same personalities, thus helps in increasing the company’s profits; and (3) time management of a person was ensured so he can be made available for multiple project assignments. MBPSO was applied to maximize team effectiveness and team efficiency, and the results of the algorithm were satisfactory [13]. However, intelligent MBPSO lost its significance when all objectives were merged into a single objective using a utility function to search for global minima [16]. In 2011, Kargar et al. [3] proposed a system for finding the team of experts with or without a leader with polynomial delay time. They considered different cost models, in which a person participates with different skills to perform a task; meanwhile, the contribution to the cost was independent for each skill. Also, their model avoids the set covering aspect and thus simplifies the problem [11].

Earlier in 2012, Aris et al. [11] considered the Lappas task assignment method an inefficient one because it only paid attention to coordination costs and ignored workload balancing among team members. Therefore, a new method of online team formation was used to find a delicate balance between workload and coordination costs so that an expert can finish multiple projects without overloading his schedule. The same year in 2012, Kargar et al. tried to answer the team formation problem by inducting personnel cost of an expert based on the number of skills he possessed. Besides personnel cost, the minimum edge connection between the experts was also considered [12, 17]. This approach created large teams that practically cannot easily incorporate the minimization of team size altogether [8]. Zhang et al. [16] argued that in order to form effective product development teams, a multi-objective particle swarm optimization (MOPSO) is required that considers all comprehensive capabilities and interpersonal relationships team members. An improved fuzzy Analytic Hierarchy Process based on fuzzy linguistic preference relation is applied to ensure the accuracy and correctness of a member’s skills. MBTI is used to model interpersonal relationships based on personality. The results of MOPSO showed that the proposed optimization model is efficient for TF.

In early 2014, Teng et al. reported the non-effectiveness of a single team leader to control team members and suggested the use of multiple team leaders to control an ever-growing team. They applied constrained communication load to limit leader communication to team members and used minimum communication cost function to create effective teams [18]. Seeing the wide possibility of creating teams in social networks, Ashenagar et al. [17] discussed two issues of team formation, i.e., the combined minimum cost of the team and the minimum time spent on team formation. In this paper, the algorithm proposed to find experts based on their closeness and eigenvector centralities. In the proposed algorithm, central experts that can reach the other nodes with minimum cost were selected based on the required skills. Central experts always select important neighbors to do other skills. If the expert’s neighbors can do other skills, the algorithm selects the minimum cost. If they do not have skills, this algorithm selects from the neighbors-neighbors central expert. The neighborhood search continues until an expert with the required skills is found. Ultimately, the algorithm finds the team with minimum cost from all candidate teams. This approach was tested on the DBLP dataset, and it accomplished less CPU time than the previous methods. Habibur Rehman et al. [19] termed TF a crowdsourcing problem in which larger groups hinder successful collaborations between members. They suggested using two factors optimization, i.e., high affinity and upper critical mass, to overcome unsuccessful collaborations in teams. The concept of high affinity was borrowed by Lappas [4], which means the experts must be comfortable or, in other words, at a close distance from each other. The use of upper critical mass was relatively novel, which effectively constrains the size of groups by splitting them into sub-groups, thus diminishing unsuccessful collaborations. Bahareh et al. [20] also tried to answer team formation problems to minimize the team’s personnel and communication costs. To an extent, their algorithm was able to reduce the overall Team formation cost.

For the first time in the data mining community in 2016, Wang et al. [7, 21] tried to introduce a framework consisting of all the previously proposed methods to form effective teams on a single platform. They effectively implemented the following TF algorithms, i.e., RarestFirst, EnSteiner, MinSD, and MinLD in C plus language. Same year Wu et al. proposed a reasonable human resource allocation through multiple team formation mechanisms. Following this mechanism, a task is based on working strength and sorted according to the contribution of agents/members in the descending edict. Ultimately, the agents who have greater contributions than others are chosen to fulfill the task [22]. Niveditha et al. proposed a Non-Dominated Sorting Genetic Algorithm (NSGA-II) based tri-objective Team formation framework to minimize communication cost, personnel cost, and cardinality the teams. Team formation in social networks was defined to produce compact, cooperative, and low-cost teams. Instead of using decade-old scalarization techniques for multi-objective problems, the NSGA-II algorithm was proposed to solve tri-objectives with affluence. The TF framework was tested on the DBLP skill and co-author dataset to obtain Pareto Optimal Solutions. The precision and recall of the obtained Pareto front to the true Pareto front generated using exhaustive search are evaluated. It was shown in the results that the NSGA-II gives compact teams that converge to the Pareto-optimal in less time [8]. Li et al. addressed maintaining and optimizing team performance in a more extensive social network against certain changes made to the team. The proposed TeamOPT worked interactively with the users to form teams with special requirements, respond to changes, and team optimization. TeamOPT was effective in finding the best candidates and provided an interactive user experience [23]. Salami et al. tried to answer the Team formation problem with an age-old metaheuristic-based Genetic algorithm (GA). Instead of using social networks of experts to answer a specific problem, experts (i.e., supervisors) interaction with the non-experts (i.e., students) for student-supervisor project allocation was presented. GA effectively allocated supervisors to students based on the fit chromosome. Besides keeping workload balance in mind, GA compared well with optimal integer programming due to the inherent advantage of producing multiple fit solutions [14]. Staden et al. also applied Team formation in digital forensics to detect the most suitable group of persons that could have committed a digital crime. This helped reduce the number of suspect groups to start the investigation, resulting in narrowing the search down to the real suspects [24].

Until 2017, all TF models tried to find people’s skills, costs of communication, personality, and other traits, but nobody tried to find reliable teams. However, Fathian et al. not only found better teams but also calculated the reliability/unreliability of a person present in a team. The team performance was further augmented by introducing backup persons if an unreliable person leaves without notice [1]. Yashar et al. redefined scientific social networks in which they defined two objectives, i.e., chemistry level (to measure the scale of communication) and expertise level (to measure the overall skills of experts filtered by chemistry level). They called their approach Chemistry Oriented Team Formation (ChemoTF) and tested on a large expertise corpus of 472,365 individual authors. The ChemoTF algorithm built more communicative and cost-effective teams with higher expertise levels [25, 26]. Taghiyareh et al. also proposed a swarm intelligent Brain Drain Optimization (BRADO) to find a team of experts in DBLP and IMDB datasets. Their results were effective PSO, GA, RarestFirst, and EnhancedSteiner algorithms [5].

The year 2018 saw several metaheuristics approaches applied in the field of TF. Baghel et al. used a genetic algorithm for creating multiple teams for different projects and a sociometric matrix for finding a positive social relationship in a TF [27]. Bagherina et al. presented a novel cat swarm-based algorithm to find the team’s communication cost and cardinality. In the proposed algorithm, each cat represents a team in the social network graph. All cats are either in seeking or tracing mode throughout the iterations until the final fit team with the minimum communication cost is found [28]. More recently, El-Ashmawi et al. proposed an improved African Buffalo (IABO) algorithm for Team formation in social networks. The IABO algorithm is unified with discrete crossover operator with swap sequence to generate better teams that cover all the skills. For minimum cost calculation among the experts, the Jaccard distance formula is used. IABO generated a team for maximum skills of 10 on DBLP and Stack Overflow datasets successfully [29]. Although IABO was quite efficient in finding teams on ten skills, large enterprises require large skills-size and teams. Although, it would have been better; if IABO was tested on more skillsets. In early 2019, El-Ashmawi again tried to answer the problem of TF with a particle swarm optimization (PSO) and the same old swap operator [2]. This time the skillsets were increased to answer large enterprise requirements, but no heed was paid to enhance the team performance other than just the minimum-cost calculation.

The year 2020 brought several advancements in the field of team formation algorithms. Earlier in 2020, Kouvatis et al. proposed a team formation signed network (TFSN) algorithm for effective communication among many individuals in a social network. They tackled the team formation problem differently than previous research by assuming that not all connections in a social network are effective. Two people can be foes or friends depending on the kind of communication they have (i.e., positive or negative). This leads them to build a signed network for two compatible individuals who can perform a task with the least communication cost. TFSN algorithm was effective on medium-sized datasets, but it was not tested on several datasets [30]. The primary goal of team formation is to utilize collective team efforts to achieve any task. Alqahtani tried to find biasness against minorities in a team formation algorithm that incorporates demographic information of an individual. The proposed diversity ranking algorithm considers race or gender during the formation of teams with minimum cost. The proposed algorithms were tested on a real dataset and produced teams with more diversity [31]. Although their work was commendable, big organizations primarily do not consider demographics for hiring a skilled individual. In early 2020, Abdulkader et al. adopted the Jaya algorithm for team formation problems in expert collaborative networks.

Jaya offers intrinsic non-parametric tuning, and it always avoids the worst solutions, thus offering global best solutions. The Jaya was tested against a state-of-the-art Sine-Cosine algorithm on an ACM dataset containing experts and their skills. The results indicate that Jaya is a reliable team formation algorithm than the Sine-Cosine algorithm [32]. The same year, Walaa H. El-Ashmawi, minimized the communication cost among skilled individuals in a team with an improved Jaya optimization algorithm. The improved Jaya algorithm used a single-point crossover swap operator to speed up the search process while minimizing the team formation problem. The proposed algorithm was tested on two real datasets and compared with genetic and other algorithms. The results show that the proposed algorithm found effective teams with minimum communication cost [33]. Seeing the unreliable nature of individuals leaving teams and causing recurrent losses to the organization, multiple team formation problems (MTFP) was proposed by Campelo. MTFP utilizes integer linear programming to group individuals into a social network of teams. For individuals, time fractions were created to facilitate him to work on different teams. MTFP was highly reliable in finding multiple teams tested on real-world social networks [34]. The major contributions to team formation (TF) in literature are given in Table 2.

**Table 2:** Popular team formation algorithms.

Algorithm (s)	Communication Cost	Personal Cost	Workload Balancing	Unique Experts	Team Reliability
[2, 4, 5, 7, 21–23, 29, 32, 33]	√				
[1]	√	√			√
[3, 16, 18, 19, 25, 26, 28, 31]	√			√	
[8, 12]	√	√		√	
[11]	√		√		
[13, 14]			√		√
[20]	√	√			
[24]				√	
[27, 30, 34]	√				√

Despite providing several optimized solutions to the TF-problems, previous researchers didn’t try to overcome the problems associated with the datasets being utilized or the CPU time offered by the algorithms. TF deemed an NP-hard problem, this paper will try to overcome both of these problems and will try to converge in polynomial time. The proposed SSR-TF algorithm is discussed in the next section.

## The proposed SSR-TF algorithm

**Fig 3:**
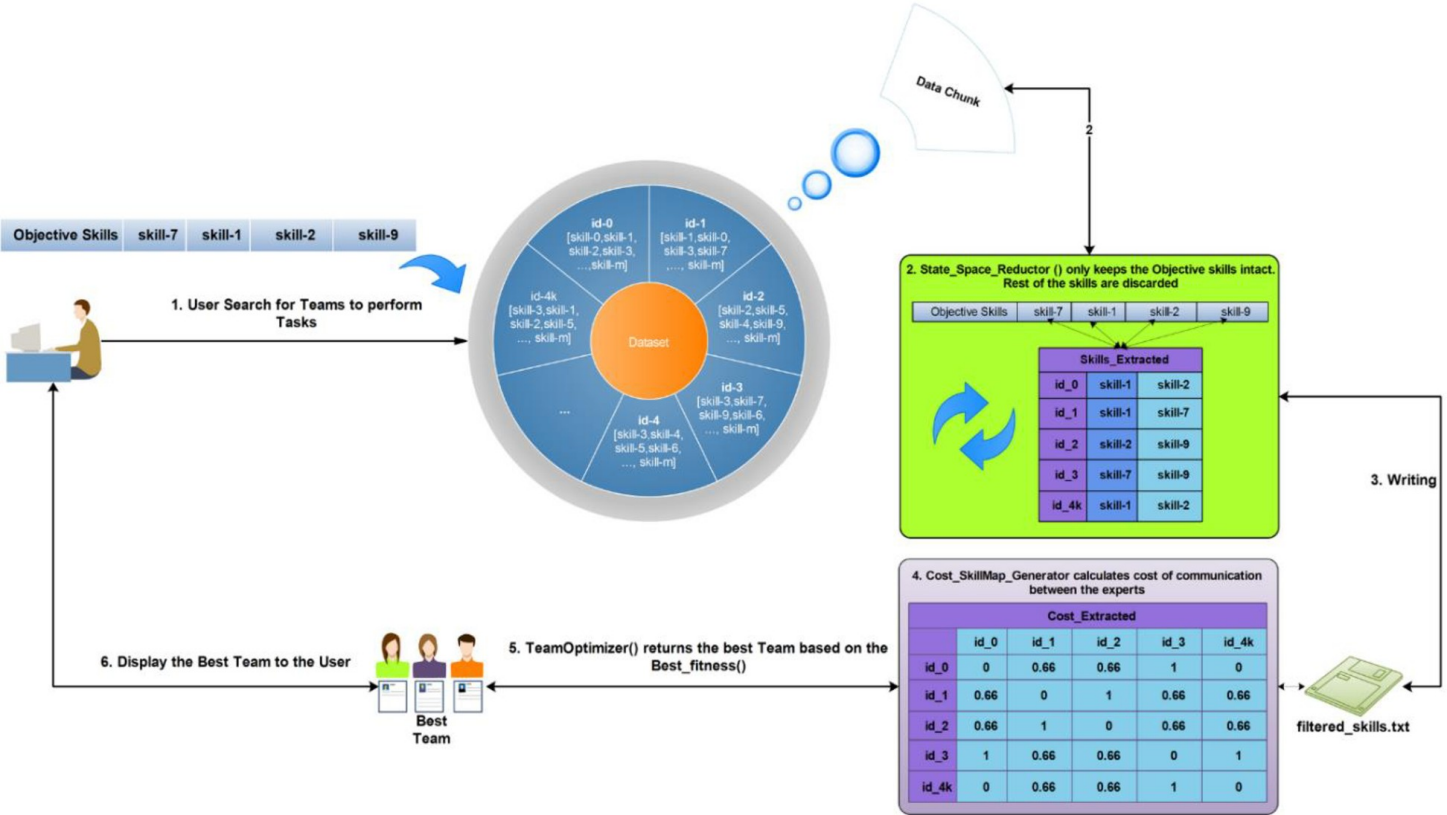
The basic architecture of team formation of experts in social networks.

Search Space Reduction-Team Formation (SSR-TF) is an entirely different approach towards solving the TF problem than the previous algorithms. Instead of entirely relying on communication-cost calculation first, this algorithm tries to reduce the features in the graph to only the appropriate ones, so there is nothing left insignificant in the data. This starts with the extraction of skills in the given task and selecting experts related to those specific skills from the dataset and then the sub-graph is formed. This step leads us towards the formation of teams with significantly lower communication costs and team members in real-time. The SSR-TF methodology is illustrated in Fig 3.

**Fig 4:**
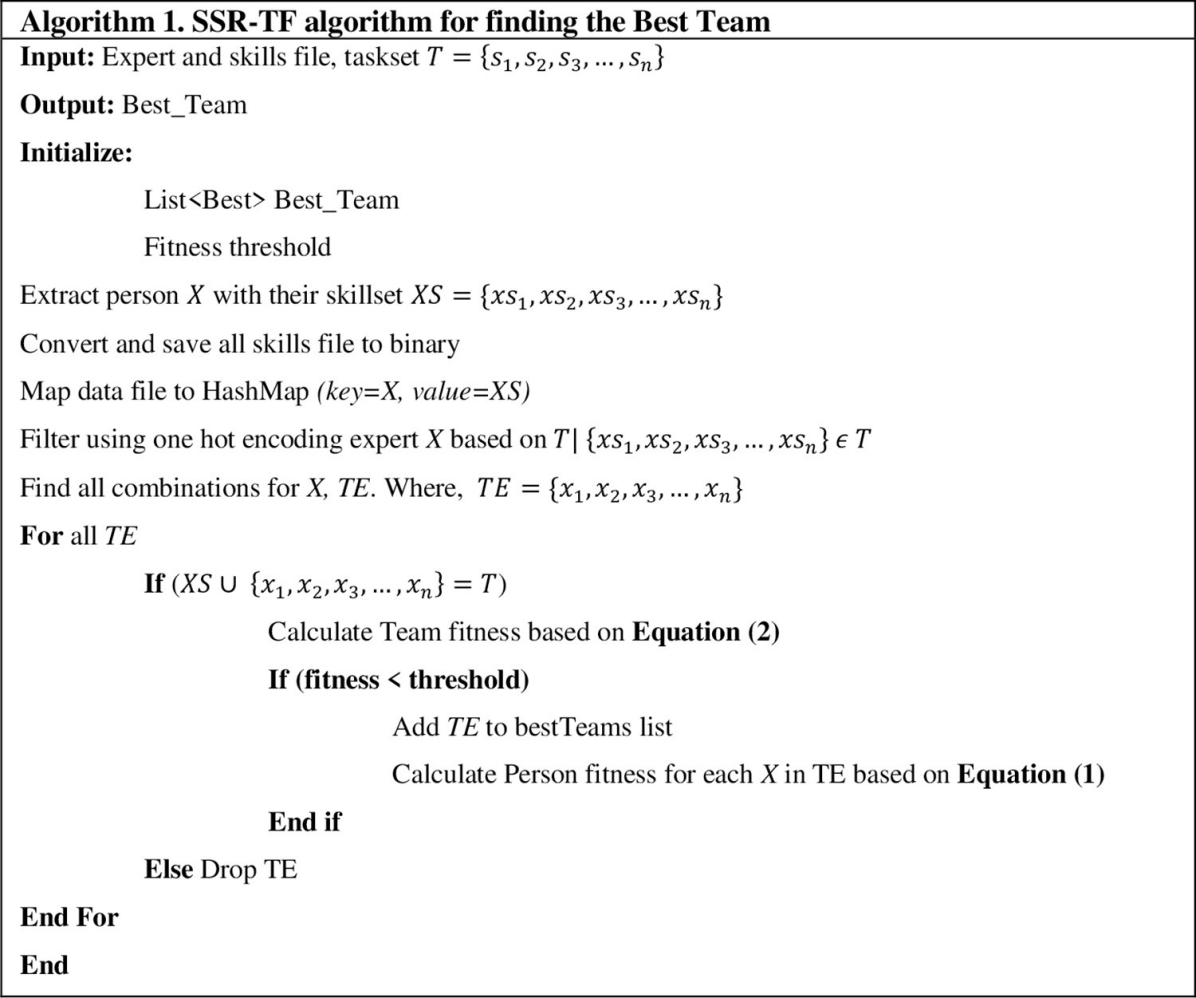
SSR-TF algorithm for finding the best team.

Using social network Graph, G, and a task T, SSR-TF builds a network in which each expert has at least one skill. Then, all the expert data is converted into binary form for faster execution. HashMap is used for linking experts with their skills. Then, one-hot encoding is applied to filter out those skills/experts which are not required, resulting in a sub-graph G’. At that time, SSR-TF starts on G’ and continues to finds all successful combinations of experts with skills. The team’s fitness is checked at each iteration with Eqs (1) and (2). SSR-TF continues to create/drop teams until the threshold level is reached or the team with the best fitness value and required skills are reached. Fig 4 shows the SSR-TF algorithm for finding the best team.

### Time complexity of the SSR-TF algorithm

The time complexity of the proposed algorithm refers to characterize the execution time, regardless of the hardware, programming language, and compiler used for implementation. This time complexity analysis evaluates the execution time variation of the proposed algorithm based on the input data size. Typically, the time complexity of such an algorithm is denoted by the asymptotic notation *(O)*. The proposed algorithm has two main searching criteria that are vertical and horizontal searches. Each search has a complexity of *nlogn*, where n is the number of individuals in the data set during the vertical search.

In contrast, *n* represents the number of searched expertise during the horizontal search. In such a case, the searching complexity of the proposed team formation algorithm is *2nlog(n)*. After searching for required individuals and their skills, there is an addition of individuals to the merger in a team that has complexity equal to the array addition that is *O(n)*. Finally, the overall time complexity of the proposed team formation algorithm becomes *O(2nlog (n*^*2*^*))*, which is comparatively less than the other approaches.

## Experiments and results

### Preliminaries

In order to demonstrate the efficiency of the proposed SSR-TF algorithm was tested on five datasets, i.e., UMP, DBLP, ACM, IMDB, and Bibsonomy. The simulation experiments were performed on an Intel Core i5 processor with 8 GB of RAM, using Java Eclipse software and Microsoft Windows 10. The proposed SSR-TF algorithm was compared with the most recent state-of-the-art metaheuristic Hill-Climbing TF, Jaya-TF [33], and Sine-Cosine-TF [32] algorithms. The selected performance parameters for team formation are Total Communication-Cost (TC), CPU time in milliseconds, and experts in a team. For all experiments, best tuning parameters are used. The datasets and their statistics are given in Table 3. Also, all the algorithm’s parameter settings are given in Table 4.

**Table 3:** Skills datasets and their properties.

Dataset	No of Experts	Overall Skill size
UMP dataset (D01)	96	164
DBLP dataset (D02)	5641	3853
ACM dataset (D03)	3856	5266
IMDB dataset (D04)	1021	27
Bibsonomy dataset (D05)	6990	19856

**Table 4:** Parameter setting of the algorithms.

Algorithm (s)	Parameter (s)
SSR-TF	max_fitness_evaluation = 1000
Hill Climbing-TF	population_size = 10
Jaya-TF [33]	max_fitness_evaluation = 1000
	max_iteration = 30
	population_size = 10
Sine Cosine-TF [32]	max_fitness_evaluation = 1000
	max_iteration = 30
	population_size = 10

#### Universiti Malaysia Pahang (UMP) dataset (D01)

Universiti Malaysia Pahang (UMP) dataset (D01) is a medium-size dataset that contains comprehensive information about 96 academicians with 164 skills related to the computer science field. It was collected by Kamal et al. [32] to find successful collaborating teams within the faculty of computing, UMP to run cost-effective projects. This team formation dataset is one of the cleanest available online [35]. A single instance of the dataset is available in the following manner “***kamalz@ump.edu.my = Combinatorial Testing*, *Computational Intelligence*, *Artificial Intelligence”*** and normalized using one-hot encoding in SSR as “***kamalz@ump.edu.my = 1 1 1 0 0 0 0”*.**

#### Database Systems & Logic Programming (DBLP) dataset (D02)

The DBLP dataset has the largest number of experts from the Database, Theory, Data-mining, and Artificial Intelligence fields. In this dataset, people having more than one paper indexed on DBLP are selected as experts. The skills of each expert are based on the title of the authored paper broken down into meaningful words. The dataset is available online [36].

#### Association for Computing Machinery (ACM) dataset (D03)

It is another dataset collected by Prof. Min-Yen Kan from the National University of Singapore. The dataset was extracted from papers published between 2003 to 2010. The authors of the paper are considered experts, and keywords are considered their unique skills. The dataset can be found online [37].

#### Internet Movie Database (IMDB) dataset (D04)

The dataset (D04) extracted from Internet Movie Database (IMDB) is quite dense than the other datasets and can test the scalability of an algorithm getting tested [7]. The dataset is collected from the year 2000 to 2002, and only those actors are considered experts who have appeared in at least eight movies during this period. The acting skills of an actor are justified by the number of genres he can perform. The communication cost of two experts is calculated with Eq (1). The dataset is normalized in the same manner as other datasets so that one algorithm can be tested on several datasets. The dataset can be downloaded here [38].

#### Bibsonomy dataset (D05)

The dataset (DO5) is a large dataset taken by Bibsonomy that provides sharing and bookmarking of scientific publications online [21]. The authors of the bookmarked publications are considered experts, and bookmarks are considered their expertise.

### Statistical evaluation of the SSR-TF algorithm

The experimental results of the proposed SSR-TF with the Hill-Climbing TF, Jaya-TF, and Sine-Cosine-TF algorithms for each skillset are discussed in the sub-sections.

#### SSR-TF and parallel metaheuristics on D01 dataset

**Fig 5:**
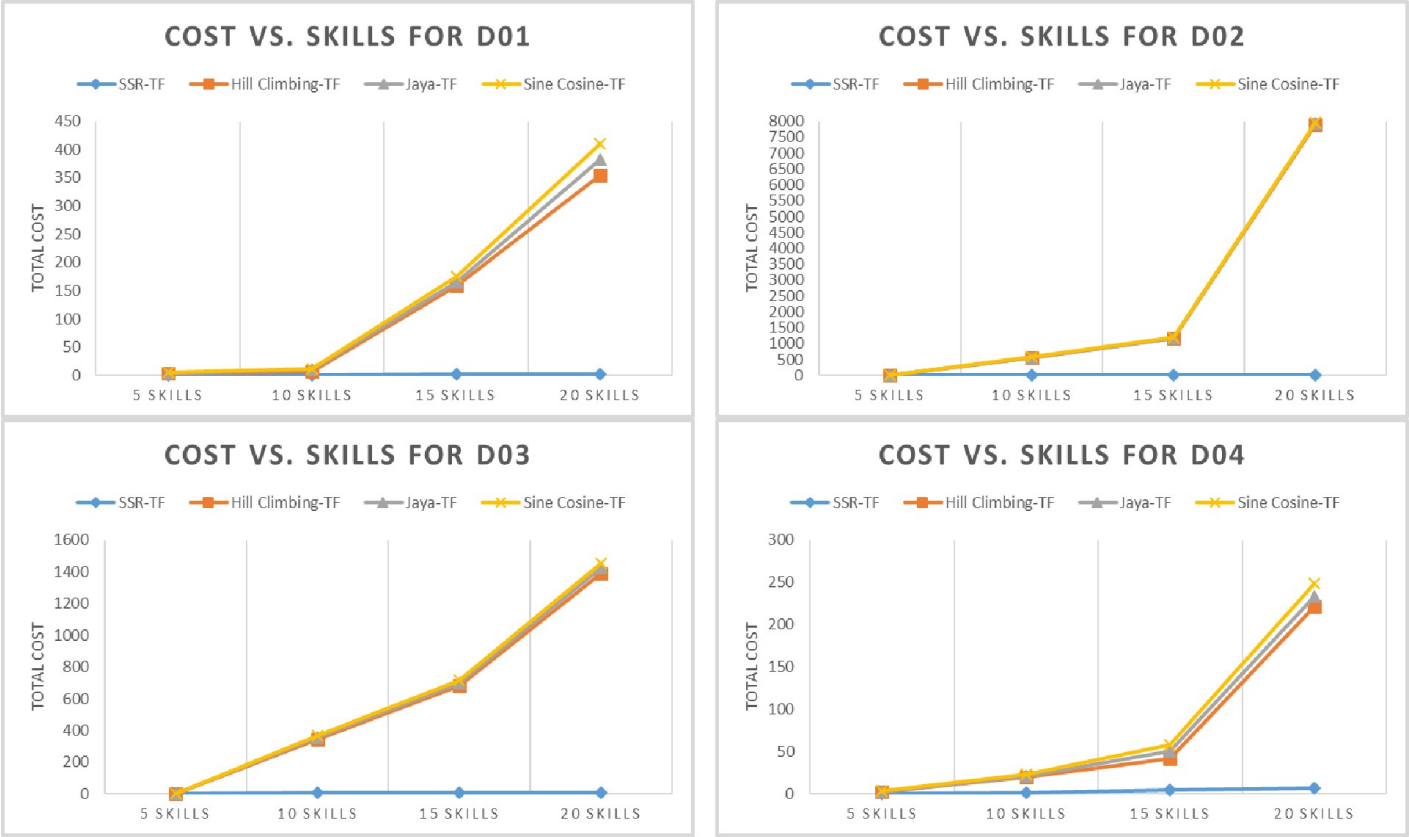
Cost performance of the algorithms on the datasets (D01-D04).

**Fig 6:**
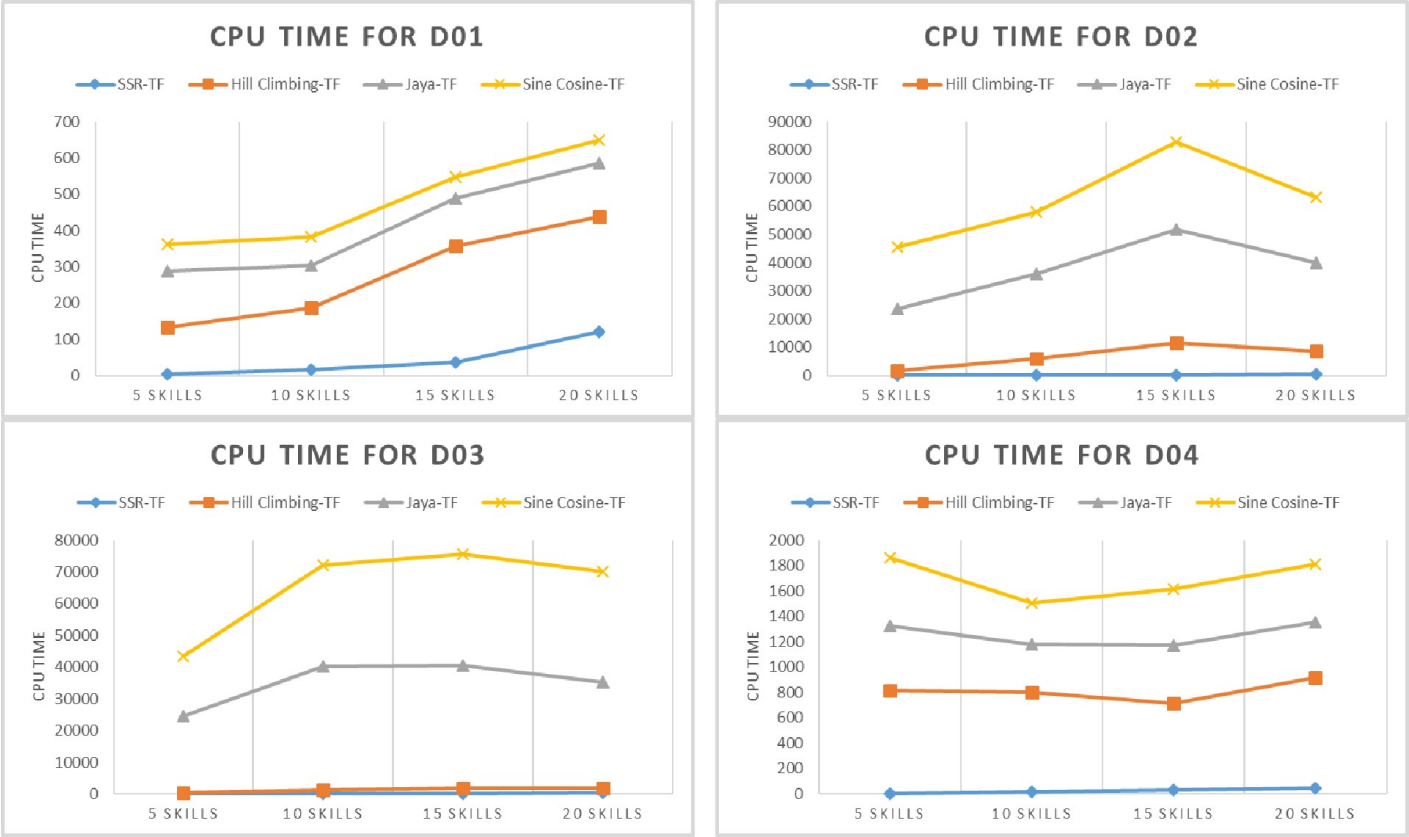
Elapsed time (in milliseconds) of the algorithms on the datasets (D01-D04).

**Fig 7:**
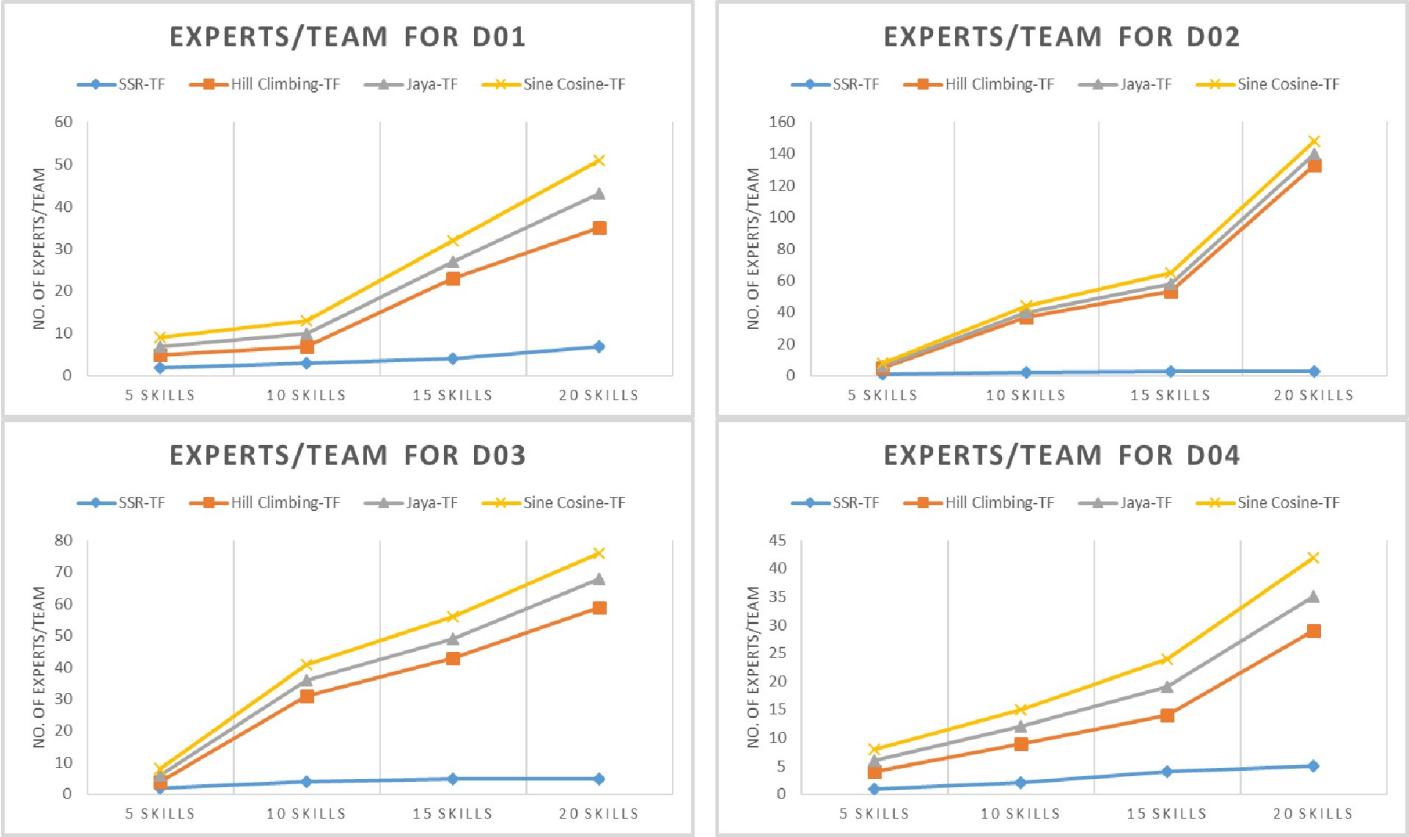
Number of experts/team selected by the algorithms on the datasets (D01-D04).

The proposed SSR-TF efficiency is tested on an organic UMP (D01) dataset against state-of-the-art metaheuristic algorithms, i.e., Hill Climbing-TF, Java-TF, and Sine Cosine-TF. The results of SSR-TF for total communication cost, elapsed time, experts, and a varying number of skills, *S* = {5,10,15,20} are given in Tables 5–8. Minimum cost vs. skills, elapsed time vs. skills, and experts vs. skills are given in Figs 5–7 (D01), respectively. For five skills, D01 was not able to find the minimum communication cost. However, its elapsed time was relatively low, as given in Table 5. The number of experts identified was the same as Jaya-TF and Sine Cosine TF. Nevertheless, as the number of skills was increased, the proposed SSR-TF started showing the best communication cost, CPU time, and number of experts. The superior result of SSR-TF for D01 with *S* = {10,15,20} is evident in Tables 5–8.

**Table 5:** Algorithm’s performance on datasets (D01, D02, D03, D04, & d05) for skillset (05).

Algorithm (s)	Total Communication-Cost					Total Time (In milli-seconds)					Number of Experts				
	D01	D02	D03	D04	D05	D01	D02	D03	D04	D05	D01	D02	D03	D04	D05
**SSR-TF**	2	0	1	0	1.39	4	30	43	07	13	2	1	2	1	2
**Hill Climbing-TF**	2.83	5.85	1	2.5	9.93	130	1827	316	812	121	3	4	2	3	5
**Jaya-TF**	1	0	1	0.4	5.76	154	21777	24178	503	200241	2	1	2	2	4
**Sine Cosine-TF**	1	0.86	1	0.64	5.78	73	21869	18997	540	200502	2	2	2	2	4

**Table 6:** Algorithm’s performance on datasets (D01, D02, D03, D04, & D05) for skillset (10).

Algorithm (s)	Total Communication-Cost					Total Time (In milli-seconds)					Number of Experts				
	D01	D02	D03	D04	D05	D01	D02	D03	D04	D05	D01	D02	D03	D04	D05
**SSR-TF**	3	0.9	6	2	2.71	16	120	154	20	57	3	2	4	2	3
**Hill Climbing-TF**	5.61	568.3	340.93	18.2	20.36	171	5795	1277	780	121	4	35	27	7	7
**Jaya-TF**	2.87	2.81	9.75	0.51	14.60	117	30183	38689	378	170318	3	3	5	3	6
**Sine Cosine-TF**	2.87	5.61	9.85	2.32	5.78	79	21785	32119	327	176187	3	4	5	3	6

**Table 7:** Algorithm’s performance on datasets (D01, D02, D03, D04, & D05) for skillset (15).

Algorithm (s)	Total Communication-Cost					Total Time (In milli-seconds)					Number of Experts				
	D01	D02	D03	D04	D05	D01	D02	D03	D04	D05	D01	D02	D03	D04	D05
**SSR-TF**	6	2.612	10	4.33	20.75	38	197	279	34	90	4	3	5	4	6
**Hill Climbing-TF**	156.8	1168.4	672.1	37.55	53.55	318	11562	1619	681	133	19	50	38	10	11
**Jaya-TF**	5.87	9.02	14.87	8.42	27.08	132	40023	38744	454	247152	4	5	6	5	8
**Sine Cosine-TF**	9.71	20.17	20.35	8.21	27.18	60	30906	34956	444	212705	5	7	7	5	8

**Table 8:** Algorithm’s performance on datasets (D01, D02, D03, D04, & D05) for skillset (20).

Algorithm (s)	Total Communication-Cost					Total Time (In milli-seconds)					Number of Experts				
	D01	D02	D03	D04	D05	D01	D02	D03	D04	D05	D01	D02	D03	D04	D05
**SSR-TF**	21.35	2.779	10	6.43	31.24	121	372	330	50	115	7	3	5	5	8
**Hill Climbing-TF**	351.81	7900.84	1382.34	214.83	88.66	318	8445	1564	865	165	28	130	54	24	14
**Jaya-TF**	27.35	20.21	34.63	11.91	53.13	147	31058	33459	436	230143	8	7	9	6	11
**Sine Cosine-TF**	27.56	27.09	27.16	15.36	63.45	63	23446	34757	460	188083	8	8	8	7	12

#### SSR-TF and parallel metaheuristics on D02 dataset

The proposed SSR-TF efficiency is evaluated on a benchmark DBLP (D02) dataset with state-of-the-art metaheuristic algorithms. The comparison results of SSR-TF are given in Tables 5–8. Minimum cost vs. skills, elapsed time vs. skills, and experts vs. skills are given in Figs 5–7 (D02), respectively. SSR-TF showed a similar communication cost as Jaya-TF, i.e., 0, but the CPU time was relatively low compared to Jaya-TF. However, both algorithms were able to identify a single expert for the same skills. Again, as the number of skills was increased, the SSR-TF started producing better results than other algorithms.

#### SSR-TF and parallel metaheuristics on D03 dataset

The proposed SSR-TF efficiency is verified on an organic ACM (D03) dataset against state-of-the-art metaheuristic algorithms. As evident from Tables 5–8, the proposed algorithm performed similarly to Jaya-TF and Sine Cosine TF. However, as the number of skills increases, SSR-TF began to generalize well on finding experts with less communication cost and time than other comparison algorithms. Minimum cost vs. skills, elapsed time vs. skills, and experts vs. skills for SSR-TF and comparison algorithms are given in Figs 5–7 (D03), respectively.

#### SSR-TF and parallel metaheuristics on D04 dataset

The proposed SSR-TF results are confirmed on IMDB (D04) dataset against state-of-the-art metaheuristic algorithms, i.e., Hill Climbing-TF, Java-TF, and Sine Cosine-TF. The results of SSR-TF are given in Tables 5–8. Unlike other datasets for skills 5, 15, and 20, the proposed SSR-TF could lead other algorithms with communication cost and time, but for ten skills, Jaya-TF performed better in total cost. The results are illustrated in Figs 5–7 (D04), respectively.

#### SSR-TF and parallel metaheuristics on D05 dataset

The performance of the proposed SSR-TF is verified on Bibsonomy (D03) dataset against other metaheuristic algorithms. As evident from Tables 5–8, the proposed SSR-TF algorithm performed better on skills 5, 10,15, & 20. As the number of skills increases, SSR-TF began to generalize well on finding the minimum number of experts with the most skills in less CPU time. Minimum cost vs. skills, elapsed time vs. skills, and experts vs. skills for SSR-TF and comparison algorithms are given in Figs 5–7 (D05), respectively.

### Non-parametric test analysis

In this paper, the Wilcoxon rank-sum test is used to determine the significance of the communication cost obtained by the proposed SSR-TF over other algorithms [39]. Wilcoxon determines hypothesis *h*_*0*_: *that all algorithms perform the same versus the alternative hypothesis*, *h*_*1*_: *that at least one algorithm is significantly better than the others*. The test is performed by considering the best communications cost obtained by the proposed SSR-TF and the parallel algorithms.

The test is conducted on the best solution obtained by each algorithm on each dataset with a 95 percent significance level (α = 0.05). In Table 9, the positive (+) sign specifies that the proposed algorithm is better than the parallel algorithm. The negative (-) sign specifies that the proposed algorithm is inferior to the compared one. As shown in Table 9, the proposed SSR-TF algorithm seems to obtain statistically significant performance than the other parallel algorithms most of the time.

**Table 9:** Wilcoxon rank-sum test results for SSR-TF against other algorithms (*α* = 0.05).

Algorithm (s)	D01	Win	D02	Win	D03	Win	D04	Win	D05	Win
**SSR-TF vs. Hill Climbing-TF**	1.60E-10	+	2.35E-13	+	1.54E-17	+	1.61E-10	+	1.23E-04	+
**SSR-TF vs. Jaya-TF**	3.11E-05	+	5.04E-01	-	4.58E-01	-	1.23E-04	+	8.21E-04	+
**SSR-TF vs. Sine Cosine-TF**	3.07E-05	+	5.04E-01	-	4.50E-01	-	1.23E-04	+	9.12E-04	+

### Threats to validity

The proposed SSR-TF algorithm has been proved to achieve better results than the other considered approaches, but there are still a few drawbacks/threats that are worth attention to be solved in the near future. In team formation research, different threats are addressed during the experimentations and evaluations. Normally, these threats are classified into internal and external. Depending on the type of research, this study is also not devoid of these threats. External threats to validity occur when the algorithm cannot generalize the experiments to the real-world problems. Mostly, the adopted benchmarks do not represent the real-world applications with the same parameters, values, and interaction strength. This threat is eliminated by choosing the most commonly used experimental benchmarks in the literature. These benchmarks are commonly used for practical evaluations and obtained from a real configurable software. Internal validity threats occur due to the factors that directly or indirectly affect the experiments and are out of control. Some of the threats to internal validity are population size, number of iterations, and parameter settings of algorithms. Besides obtaining best results, mean results are used to ensure robust performance on each algorithm. Generation time for each algorithm also threatens the internal validity. Running environments, data structures, implementation languages, and the operating environments highly effects the generation time. This threat was eliminated by implementing all algorithms in the same language and operating environments. SSR algorithm is tested on clean and middle-sized datasets containing less complex and low volume instances, which does not give the behavior of this approach on high volume and complex datasets. The algorithm also contains the string to binary and binary to string conversions, which is an additive process other than the actual working of the algorithm. Less complex data transformation methods can replace this dual conversion process of data.

## Conclusions and future works

Team formation (TF) in social networks uses the graph search to provide collaboration between experts. This led us towards forming cost-effective research teams irrespective of the geolocation of the experts and the size of the dataset. Several TF-formation algorithms were proposed in the past decade, but they failed to scale well on large datasets. Therefore, this paper presents a novel TF algorithm for expert team formation called SSR-TF based on two metrics; communication cost and graph reduction, that will become a basis for future TF’s. The decades-long efforts to produce cost-effective teams in social networks that can converge in polynomial time are successfully achieved with SSR-TF. SSR-TF has efficaciously created social teams of experts and showed its prowess when tested against state-of-the-art metaheuristic Hill-Climbing TF, Jaya-TF, and Sine-Cosine-TF algorithms. The reduced graph feature of SSR-TF has enabled to selection most appropriate experts with the proper skills to finish a task. Besides offering benefits like appropriate person selection and polynomial time, SSR-TF has opened new future horizons for the researchers towards creating teams in a number of ways;

- SSR-TF performance will be enhanced with the introduction of personal cost for each expert based on the years of experience, task/project leader selection based on the number of skills for leading a specific project team, and identifying backup teams in case the leading team’s personnel are missing or unable to finish the task.
- Sometimes, global collaborations require more skills to be handled by the team, therefore in the future, SSR-TF will be tested on a large number of skills against other metaheuristic algorithms.
- The current COVID’19 pandemic and the death toll it caused led us to believe that we should be prepare for any future pandemics. The preparedness to stop any future pandemics can be ensured by creating an expert dataset of virology and other diseases. So, when an outbreak occurs, TF can be applied to gather brilliant minds from all over the globe and solve the problem effectively.
